# Depletion of MHC supertype during domestication can compromise immunocompetence

**DOI:** 10.1111/mec.15763

**Published:** 2020-12-22

**Authors:** Willow Smallbone, Amy Ellison, Simon Poulton, Cock van Oosterhout, Joanne Cable

**Affiliations:** ^1^ School of Biosciences Cardiff University Cardiff UK; ^2^ School of Biological Sciences University of East Anglia Norwich Research Park Norwich UK; ^3^ School of Environmental Sciences University of East Anglia Norwich Research Park Norwich UK

**Keywords:** domestic animals, *Gyrodactylus*, inbreeding, major histocompatibility complex, ornamental, *Poecilia reticulata*

## Abstract

The major histocompatibility complex (MHC) plays an important role in infectious disease resistance. The presence of certain MHC alleles and functionally similar groups of MHC alleles (i.e., supertypes) has been associated with resistance to particular parasite species. Farmed and domesticated fish stocks are often depleted in their MHC alleles and supertype diversity, possibly as a consequence of artificial selection for desirable traits, inbreeding (loss of heterozygosity), genetic drift (loss of allelic diversity) and/or reduced parasite biodiversity. Here we quantify the effects of depletion of MHC class II genotype and supertype variation on resistance to the parasite *Gyrodactylus turnbulli* in guppies (*Poecilia reticulata*). Compared to the descendants of wild‐caught guppies, ornamental fish had a significantly reduced MHC variation (i.e., the numbers of MHC alleles and supertypes per individual, and per population). In addition, ornamental fish were significantly more susceptible to *G. turnbulli* infections, accumulating peak intensity 10 times higher than that of their wildtype counterparts. Four out of 13 supertypes were associated with a significantly reduced parasite load, and the presence of some supertypes had a dramatic effect on the intensity of infection. Remarkably, the ornamental and wildtype fish differed in the supertypes that were associated with parasite resistance. Analysis with a genetic algorithm showed that resistance‐conferring supertypes of the wildtype and ornamental fish shared two unique amino acids in the peptide‐binding region of the MHC that were not found in any other alleles. These data show that the supertype demarcation captures some, but not all, of the variation in the immune function of the alleles. This study highlights the importance of managing functional MHC diversity in livestock, and suggests there might be some immunological redundancy among MHC supertypes.

## 1 INTRODUCTION

The major histocompatibility complex (MHC) is a multigene family that plays an integral part in raising an adaptive immune response that is unique to the jawed vertebrates (Cooper & Alder, 2006). Since their discovery in the 1970s (Thorsby, 1974), the MHC genes have been the leading candidates for infectious disease susceptibility, displaying high levels of genetic polymorphism in all but a few vertebrate populations examined to date. To account for the extreme variability observed at the MHC loci of mice (the H‐2 loci), Doherty and Zinkernagel (1975) proposed that infectious diseases were the major selective force. Since then, it has become apparent that genetic diversity at the MHC plays an important role in disease resistance to pathogens in many systems (Milinksi, 2014). Differences in MHC molecules, particularly at the peptide‐binding region (PBR), lead to variation in the ability of an individual to initiate an immune response against particular infections (Milinski, 2014). Many studies have shown that individuals with high MHC allelic diversity tend to be more resistant than those with low diversity (e.g., Arkush et al., 2002; Carrington et al., 1999; Hedrick et al., 2001; Turner et al., 2007). Other studies have found that an optimal (i.e., intermediate) level of individual MHC diversity ensures the greatest pathogen resistance (Kalbe et al., 2009; Wegner et al., 2003), although this may not always lead to a stable polymorphism (Hedrick, 2004). More recently, the presence of particular MHC alleles has been correlated with the presence or absence of a particular parasite species (e.g., Lei et al., 2016; Schwensow et al., 2007; Trachtenberg et al., 2003; Zhang et al., 2015). Phillips et al. (2018) showed that novel alleles offer a superior parasite resistance. Various models of balancing selection have been proposed that promote high levels of MHC diversity in outbred populations, with some species possessing hundreds of alleles per locus (Lighten et al., 2017).

To simplify the bewildering allelic richness of the MHC, researchers have grouped alleles in functionally equivalent clusters, known as supertypes (Sette & Sidney, 1999; Sidney et al., 1996). The various clustering algorithms are based on inferred shared functional properties of the amino acids of the PBR (Doytchinova & Flower, 2005; Doytchinova et al., 2004). Classification of MHC alleles into supertypes has been valuable in vaccine development, by targeting specific groups of antigen epitopes (Sette et al., 2002; Sette & Sidney, 1998; Sidney et al., 1996). Surprisingly few studies have, however, focused on the association of MHC supertypes with parasite intensity (exceptions include: Fraser & Neff, 2010; Pilosof et al., 2014; Schwensow et al., 2007). To the our knowledge, no study has compared the supertype variation between wild and domesticated stocks, or assessed the impact of possible changes in MHC variation of captive‐bred populations on parasite resistance.

The farming and domestication of animals tends to lead to a reduction in morphological, behavioural and physiological trait diversity (e.g., Geiser & Ferguson, 2001; O’Regan & Kitchener, 2004; Price, 1999). Domestication of animals can reduce genetic variation directly when a particular genetic variant is selected for in the artificial environment. Indirectly, artificial selection can also erode genetic variation around the genes favoured during the domestication process, in a phenomenon known as a selective sweep. Furthermore, given that the actual breeding stock of many domesticated animals is often relatively small, genetic polymorphisms may be lost due to drift. Besides the direct and indirect effects of artificial selection, and the effect of genetic drift, there is yet another effect that particularly impacts the polymorphism at immune genes in captive populations. Such populations tend to encounter reduced parasite diversity due to monoculture and husbandry practices, such as the regular cleaning of captive facilities and pathogen control programmes, including the widespread use of antibiotics (Buehler et al., 2008; Friend & Franson, 1999; Joop & Rolff, 2004). Limited exposure to a range of parasites is likely to weaken the strength of (parasite‐mediated) balancing selection, potentially resulting in the loss of immunogenetic polymorphisms. This may be critical for genes with exceptionally high levels of polymorphism such as the MHC, which are likely to suffer from the gradual loss of allelic variation and supertype diversity over time, in a phenomenon known as the “drift debt” (Gilroy et al., 2017). There have, however, been few comparisons of immune function in captive‐bred animals and (recently collected) wildtype individuals (e.g., Abolins et al., 2011; Boysen et al., 2011; Buelher et al., 2008; Devalapalli et al., 2006; Pi et al., 2015; Viney et al., 2015).

Guppies (*Poecilia reticulata*) provide an exceptional model for investigating the impact of domestication on immune‐competence. They occur in the wild in a range of different habitats and have invaded all continents, with the exception of Antarctica. In addition, ornamental guppy populations have been artificially selected worldwide in the aquarium trade over many generations (>100) to produce ~ 100 strains with distinct colour patterns, fin size and body size (Dykman, 2012). Consequently, ornamental guppy strains have significantly reduced diversity at neutral genetic markers (Bleakley et al., 2008; Shen et al., 2007). Guppies are thought to possess a variable number of MHC class IIB loci, ranging between one and four (van Oosterhout et al., 2006). First studied by Sato et al. (1995), guppy MHC loci have high allelic variation, which differs significantly between wild populations in Trinidad and Tobago (Fraser & Neff, 2010; Lighten et al., 2017; van Oosterhout et al., 2006). Captive‐bred guppy strains show a reduced diversity of MHC alleles compared to their wild conspecifics (van Oosterhout et al., 2006), but the impact on MHC supertype variation and the resulting effects on their immunocompetence remain unknown.

Particular MHC polymorphisms in guppies may be associated with defence against common *Gyrodactylus* spp. (viviparous monogenean parasites) (Fraser & Neff, 2010). Parasites play an important, yet relatively under‐investigated role in the ecology and evolution of guppies in their native environment in Trinidad, Tobago and South America (Cable, 2011). A recent study by Mohammed et al. (2020) analysed the parasite fauna of 270 guppies in 18 populations in Trinidad, detecting in total 21 parasite species. The parasite assemblages were dominated by digeneans (57% of all individual infections), followed by monogeneans (42% of all infections), and the most abundant parasite was *Gyrodactylus bullatarudis*, which was present in 12 populations (67%). *Gyrodactylus* species are ectoparasites that can be easily monitored over time in the laboratory, without destructive sampling (Cable, 2011). This makes them exceptional models to study host–parasite dynamics and quantify the impact of potential erosion of MHC supertype variation during domestication. The current study analyses MHC genotype and supertype variation of 11 ornamental strains of guppies, as well as guppies that are recent descendants from two Trinidadian populations, which we will refer to as “wildtype.” We compare infections with *Gyrodactylus turnbulli*, a native and common parasite of the guppy that was present in eight out of 18 populations (44%) sampled by Mohammed et al. (2020). We monitored the infections on individual hosts during 17‐day infection trials, examining the change in parasite intensity over time, on fish with known MHC class II alleles and supertypes. In addition, we analyse amino acid similarities between MHC supertypes in order to account for the different supertypes detected in wildtype and ornamental fish.

## 2 MATERIALS AND METHODS

### 2.1 Host origins and maintenance

About 300 guppies were collected from each of two large wild populations of the Lower Aripo (10°35′00″N, 61°14′00″W) and Tacarigua (10°37′00″N, 61°24′00″W) Rivers in Trinidad (Barson et al., 2009) and transported to the UK in 2012. Both brood stocks of these fish were kept in captivity for 4 years (about eight generations) at a census population size of ~300 individuals, with no exposure to parasites, including *Gyrodactylus* spp., and without selective breeding. Here, we will refer to these guppies as “wildtype,” but we note that these fish represent only two populations from a species with a global distribution, and that they may differ from guppies caught directly from the natural environment because the captive conditions in a laboratory differ from those in the wild.

Eleven strains of ornamental strains of guppy were used for this study. Eight strains were purchased from a pet shop supplier in November 2014 (Black, Blonde Red, Cobra Green, Flame, Neon Blue, Yellow German, Leopard and Sunset Blonde). These ornamental fish had undergone intense selective breeding, and were phenotypically very similar within a strain, but very distinct between strains. These strains of fish were treated for parasites on entering the laboratory as part of their quarantine, and it is likely that they had been exposed to gyrodactylid parasites, like most guppies in the aquarium trade, but we cannot be certain of their previous parasite exposure history. A ninth ornamental strain, Balcony, originated from a Nottingham pet shop in 1997 and had been maintained at Bristol (until 2000) and then Cardiff without selective breeding (until 2016); these fish had been parasite‐free since 1998. Two additional ornamental strains were purchased from Tartan Guppy (Black strain) and Frisby Aquatics (Red strain) in 2015 and 2005, respectively, and reared at Hull University before being transferred to Cardiff University in 2015.

Experimental infections were performed in 2016 on wildtype and ornamental strains of guppy selected at random for this study. All fish used for experimental infection and/or genetic analysis were maintained at Cardiff University under 24 ± 1°C and 12‐h light: 12‐h dark cycle, and fed twice daily with aquarian tropical fish flakes and weekly with frozen bloodworms.

Eight to 10 months prior to the experimental infections, fish (*n* = 1056) were anaesthetized using 0.2% MS222 and individually marked with a Visible Implant Elastomer (VIE) on either the left or rthe ight flank, above or below the spine. A fin clip taken from the caudal fin of each fish was placed into a 2‐ml Eppendorf tube with absolute ethanol (99.9%) and stored at −18°C before processing. Tools and workstation were sterilized between individual samples. In total, 762 ornamental (from 11 strains) and 294 wildtype (from two rivers) fish were fin clipped for genetic analysis; a subsample of these fish was available for infection studies.

### 2.2 Experimental infection

Fish for experimental infection (Wildtype: Tacarigua, *n* = 22; Lower Aripo, *n* = 8; Ornamental: strains: Balcony, *n* = 4; Black, *n* = 8; Blonde Red, *n* = 13; Cobra Green, *n* = 8; Flame, *n* = 4; Neon Blue, *n* = 5; Sunset Blonde, *n* = 13; and Yellow German, *n* = 10) and control group (Wildtype: Tacarigua, *n* = 17; Lower Aripo, *n* = 5; Ornamental: strains: Balcony, *n* = 4; Black, *n* = 4; Blonde Red, *n* = 6; Cobra Green, *n* = 2; Flame, *n* = 3; Neon Blue, *n* = 1; Sunset Blonde, *n* = 5; and Yellow German, *n* = 6) were kept across five identical 70‐L aquaria (mixed strain, individuals identified by VIE mark) for 5 days of acclimatization, prior to the start of the study. During this time fish were fed daily on live *Artemia*. Guppies were individually separated into 1‐L containers, where they remained isolated for the duration of the infection trial. Fish were fed live newly hatched *Artemia* every day and water was changed every second day. Fish were experimentally infected with *Gyrodactylus turnbulli* (strain Gt3), which has been maintained in a laboratory culture on guppies since October 1997, when it was isolated from a Nottingham aquarium shop (same origin as the “Balcony” fish described above). Fish were anaesthetized using 0.2% MS222 and infected with two individual *G. turnbulli* on day 1 (following standard methods of King & Cable, 2007). Fish were re‐examined 12 h later to ensure the parasites had attached; all fish retained their infection. Parasite infections were then monitored every 48 h for 17 days, when fish were anaesthetized (both controls and infected fish) and the total number of gyrodactylids was counted. Control fish were anaesthetized and sham‐infected, and were monitored at the same time as experimental fish.

### 2.3 Ethical note

All work followed ARRIVE guidelines, was approved by Cardiff University's Animal Ethics Committee and was conducted under UK Home Office Licence (PPL 302876).

### 2.4 MHC genotyping

Genetic analysis was performed on a total of 1079 guppies; 760 individuals of 11 strains of ornamental guppies (Balcony [*n* = 146], Black [*n* = 35], Blonde Red [*n* = 61], Cobra Green [*n* = 60], Flame [*n* = 39], Neon Blue [*n* = 36], Yellow German [*n* = 58], Leopard [*n* = 51], Sunset Blonde [*n* = 57], Tartan Guppy Black [*n* = 119] and Frisby Aquatics Red [*n* = 98]), as well as 284 wildtype guppies (Lower Aripo [*n* = 223] and Tacarigua guppies [*n* = 61]).

Total genomic DNA was extracted from fin clips using the Zymo Research ZR‐96 Quick‐gDNA kit. A 217‐bp fragment of MHC class IIb (encompassing all but three codons), predicted to comprise the PBR (Bondinas et al., 2007; Brown et al., 1993), was amplified using the degenerate primer pair Po_ii_2_01F, 5′‐GTTGTGTCTTTARCTCSHCTG‐3′ (Herdegen et al., 2014), and eg2inR, 5′‐ATCGGCTCACCTGATHTA‐3′ (S. Paterson, pers. comm.). These primers are able to amplify MHC class IIb alleles from multiple loci (Herdegen et al., 2014). Both forward and reverse primers had an additional 10‐bp barcode at the 5′ end to uniquely identify individual amplicons and enable highly multiplexed sequencing of samples (Roche Diagnostics Technical Bulletin TCB No. 005–2009). Polymerase chain reactions (PCRs) contained 4 µl of ~60 ng genomic DNA, 10 µl Qiagen 2× PCR Master mix, 2 µl Qiagen Q and 2 µl 0.5 μm of both forward and reverse primers. The reaction conditions were as follows: 15 min denaturation at 95°C; 40 cycles of 30 s at 95°C, 90 s at 52°C and 90 s at 72°C with a final elongation step of 10 min at 72°C before being held at 10°C.

PCR products were divided approximately equimolarly (quantified via a Qubit BR dsDNA assay) into two pools (810 samples per pool). The two amplicon pools were cleaned using a QIAquick PCR purification kit and prepared for 150‐bp paired‐end Illumina MiSeq (Illumina) sequencing using the Illumina PCR‐free TruSeq library protocol with 20% PhiX spike in. A total of 20,965,456 reads were generated. Validation of the repeated sequencing runs and the genotyping protocol was performed to ensure repeatability within and between sequencing runs, and 109 samples were amplified and sequenced twice for validation. After demultiplexing and tag identification, the mean (± *SE*) number of reads per individual was 4656.69 (±23.04).

### 2.5 Genotyping with AmpliLEGACY (Lighten)

Sequences were trimmed to allow direct comparison with previously published data (Lighten et al., 2014). Individual genotypes were determined using the automated clustering and genotyping method AmpliLEGACY Lighten (part of the AmpliSAT package, Biedrzycka et al., 2017). AmpliLEGACY Lighten uses the genotyping method described by Lighten et al. (2014), which clusters variants to decipher real alleles and artefacts before using the degree of change (DOC) method. AmpliLEGACY Lighten was run using the clustering and genotyping parameters described by Lighten et al. (2014) with maximum amplicon depth = 5000. A total of 164 individuals that had <600 reads were removed from downstream analyses. A subset of genotypes was selected at random and checked against manual interrogation, where an individual had <4 alleles and 5000 amplicon read depth there was 100% similarity between manual interrogation and AmpliLEGACY (Lighten). Discrepancies were identified when individuals had been given >4 alleles or where read depth was <5000 per individual. For all these individuals, identified by AmpliLEGACY (Lighten), the genotypes were manually checked and where necessary the genotypes were updated. This curation changed the automatic calling of 58 (3.9%) of all genotypes.

### 2.6 Supertype clustering

Alleles were clustered into supertypes using the amino acid sequences of codons previously identified as being under positive selection (Lighten et al., 2014). Amino acids were substituted for a set of five physicochemical properties (Sandberg et al., 1998). Clusters of functionally similar MHC alleles were identified based on physicochemical properties of translated amino acids inferred to comprise the PBR (Doytchinova & Flower, 2005) using a discriminant analysis of principal components, performed using the packages ade4 and adegenet (Jombart, 2008). All MHC alleles identified in the current study (*n* = 174) and also published alleles (*n* = 84, retrieved from NCBI GenBank) were included. Allele sequences were aligned using bioedit, retaining all alleles that had all 15 PBRs in the sequence.

The numbers of clusters (*k*) were identified using the method described by Phillips et al. (2018) (see also Data S1). MHC alleles were clustered into 13 supertype groups based on the amino acid sequence of the PBR where there is a drop off in rate of increase of change in the Bayesian Information Criterion (ΔBIC; Figure S1). Adding additional clusters dramatically affected ΔBIC (Figure S1). The discriminant analysis of principal components (DAPC) was performed to identify the probability of an allele being in a particular supertype (see Phillips et al., 2018). Allele sequences were assigned their modal supertype cluster. Alleles that were not consistent in their supertype clustering were removed from downstream analysis (*n* = 33, 13%). A phylogenetic tree showing the relationship between these alleles is shown in Figure S2.

### 2.7 Genetic algorithm to compare similarities between supertypes

Given that ornamental and wildtype fish differed in the supertypes that were associated with resistance to *G. turnbulli* infections (see Results), amino acids in the PBR were identified that were uniquely shared between pairs of “resistant” supertypes. A genetic algorithm that imported a matrix of 15 amino acids from the PBR (columns) for all alleles (rows) was developed. The matrix also included a column encoding the 13 different supertypes, which is the categorical variable that is set as the target. The algorithm uses a matrix of predictor variables (the 15 amino acid [AA] values) to randomly generate a set of predictive expressions (e.g., the first AA is a leucine). For each allele, this is evaluated as true or false. The algorithm can also generate compound expressions that are linked to logical operators (e.g., the first AA is a leucine, the eighth AA is not a cystine). Each predictive expression is evaluated against the target‐true expression using a penalized phi‐coefficient. The algorithm loops through the number of specified generations (default 1000). For each generation, it extracts the top *n* (user‐defined) scoring rules that identify AAs which best define commonalities between “resistant” supertypes. The code is written in Transect‐SQL, as implemented in Microsoft SQL Server version 14.0.2002.14, with a Microsoft ASP.NET front end, running as a cloud application. The logical model is given in Data S3.

### 2.8 Statistical analysis

Differences between wildtype and ornamental guppies in their MHC genotypes and supertypes were visualized through nonmetric multidimensional scaling (NMDS). The ordination was run for 1000 iterations; with stress scores of 0.04 for allele community and 0.06 for supertype community. This resulted in high‐resolution differentiation that could be interpreted and visualized in two dimensions. Differences in the allele and supertype community between wildtype and ornamental guppies were assessed using the *manyglm* function in R (Warton, 2011). This method computes the analysis of deviance for a multivariate generalized linear model (GLM), fitting a single GLM to each response variable with a common set of predictors. Monte Carlo resampling tested for a significant community‐level response to the predictors. Analysis was conducted with (i) allele and (ii) supertype community as the dependent variable being explained by host type (wildtype or ornamental) and strain of host. An anayslsis of variance (ANOVA) was used to test differences in the number of alleles and supertype per individual (*Ai* and *STi*, respectively) between wildtype and ornamental fish.

To assess the effect of supertypes on parasite intensity, a negative binomial generalized linear mixed model (GLMM) was performed. The starting model included the explanatory variables experimental day, host standard length, host allelic diversity and host supertype diversity and the dependent variable *G. turnbulli* intensity. The data were subdivided into wildtype and ornamental fish due the presence of some supertypes in only one of these host types; a significant difference between these two groups of fish was identified in the previous analysis, justifying the subsetting (Data S2). It was not possible to include all of the biologically relevant explanatory variables in the starting model without subsetting. Also, we were unable to include strain as a factor when looking at the influence of different supertypes (STs) because we do not have enough degrees of freedom in the statistical model. We therefore conduct the analysis at the level of wildtype and ornamental fish. Microsoft Excel was used to determine the binomial distribution probability (binomdist) of MHC supertypes between wildtype and ornamental fish; the results suggested a significant difference giving additional reason for data subsetting. The additional explanatory variables included for (i) ornamental fish: ST3, ST4, ST5, ST7 and ST12; and (ii) wild fish: ST3, ST4, ST5, ST7, ST12 and ST13, and the interaction between each of these supertypes and experimental day was also included in the model to identify if the supertype functional cluster is associated with parasite burden.

In addition to the population genetic analysis on the MHC, the differences in parasite infection rate between the ornamental and wildtype fish were analysed. Parasite intensity was recorded for each individual fish at different time points. Hence, “Fish ID” was included as a random effect in all of the GLMMs to avoid pseudoreplication, by incorporating repeated‐measures. The residuals of all of the models were normally distributed. Only significant terms are reported. Variation in parasite intensity, defined as the number of worms on an infected host (Bush et al., 1997), between wildtype and ornamental strains of fish was analysed using a negative binomial GLMM. The GLMM used parasite intensity as the response variable, with the explanatory variables: number of days post‐infection (days); host standard length; whether the host was a wild or ornamental strain; the interaction host standard length × wildtype/ornamental and the interaction day × wildtype/ornamental.

Statistical analyses were conducted using R version 2.15.1 (version 1.0.136, RStudio 2009–2016 RStudio, Inc.).

## 3 RESULTS

### 3.1 Parasite dynamics on wildtype and ornamental strain guppies

**FIGURE 1:**
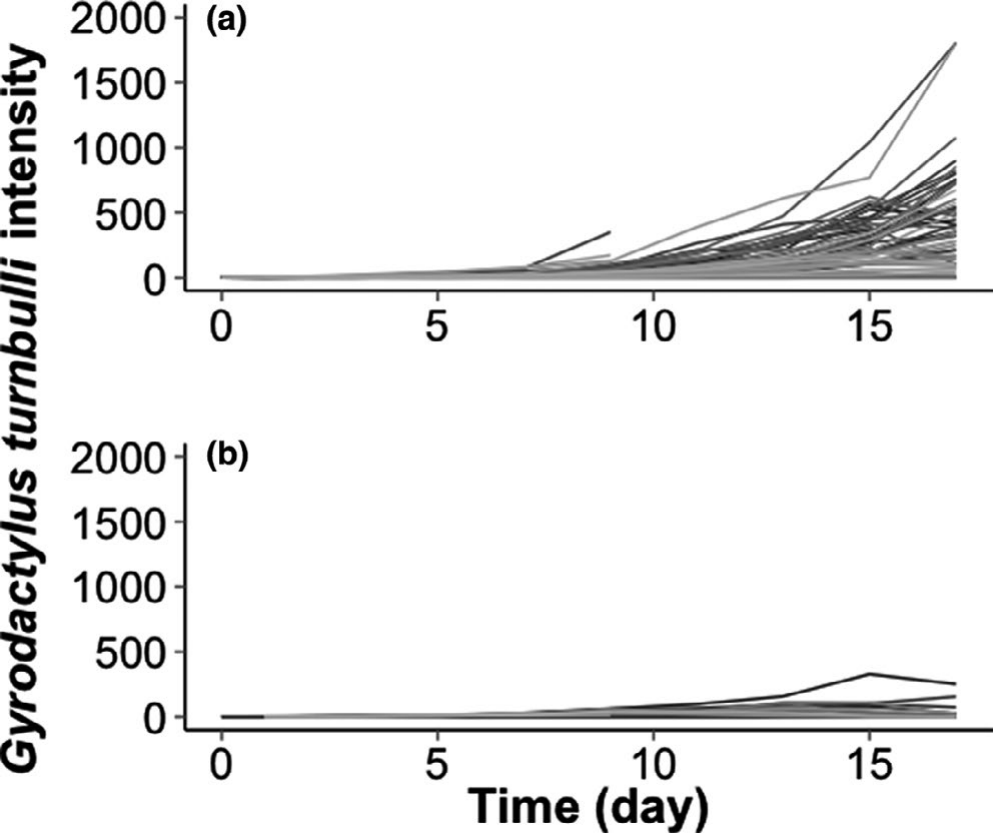
Number of *Gyrodactylus turnbulli* parasites on individual (a) ornamental (*n* = 58) and (b) wildtype (*n* = 27) guppies (*Poecilia reticulata*)

Parasite number increased significantly over time (GLMM: *z*_1747_ = 50.53, *p* ≤ .001). Over the 17‐day infection trial, ornamental fish had significantly higher parasite intensity than wildtype fish (GLMM: *z*_1747_ = −13.31, *p* ≤ .001; Figure 1). In wildtype fish, the *Gyrodactylus turnbulli* infections peaked at day 13, reaching a median of 31 worms. The infection on ornamental fish was higher at this point of the infection (median 128 worms) and infections continued to increase, reaching a median of 357 worms by day 17 (compared to a median of one worm on wild fish on day 17; Figure 1). Parasite intensity was significantly different between strains over time (GLMM: *F*_9744_ = 5.50; *p* ≤ .001; Figure S3; Table S1).

### 3.2 Host MHC allele and supertype variation

**FIGURE 2:**
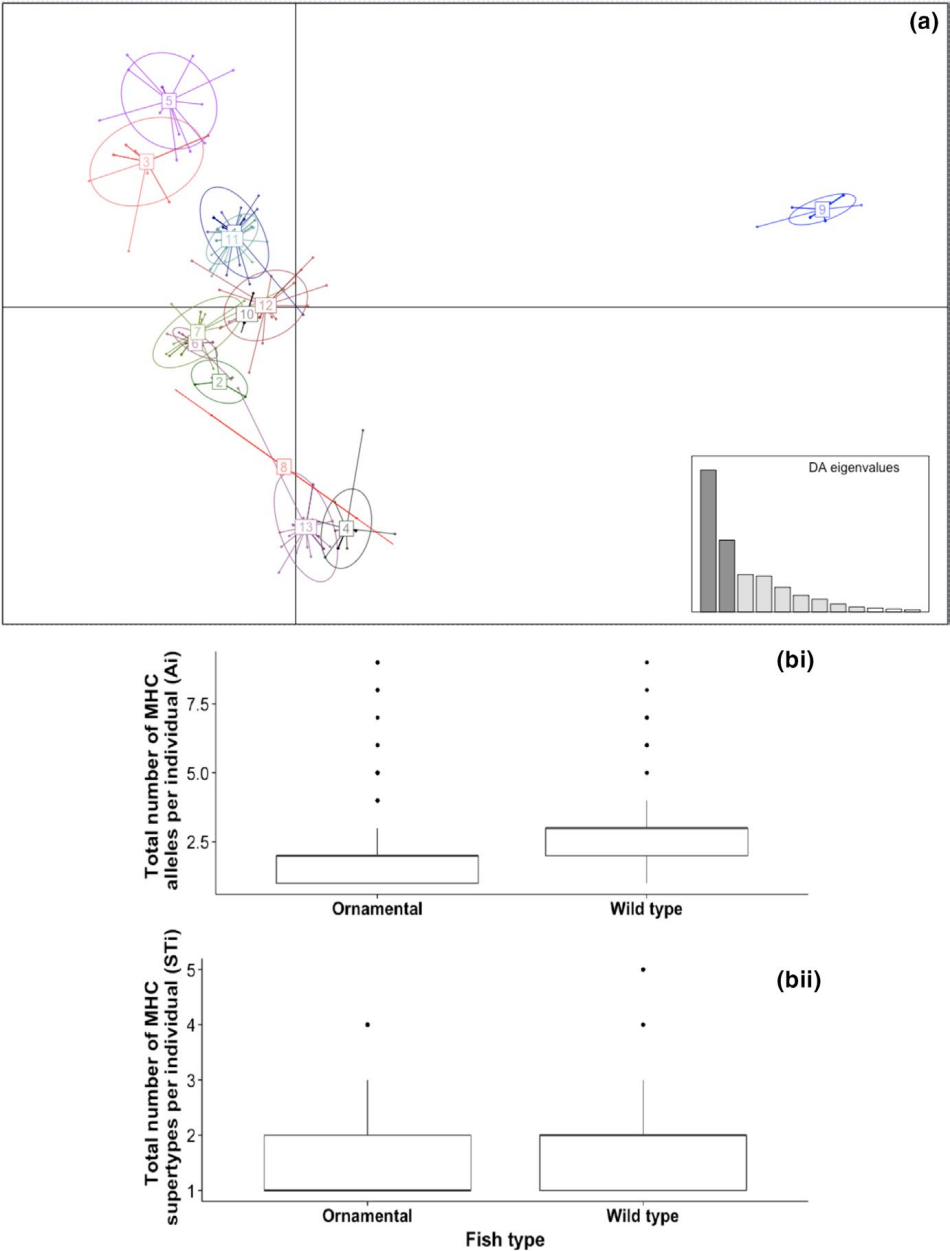
(a) Clustering of major histocompatibility complex (MHC) alleles in *Poecilia reticulata*, based on the physicochemical properties of translated amino acids inferred to comprise the peptide‐binding region using discriminant analysis of principal components (DAPC). DAPC inferred the presence of 13 clusters, or supertypes, which are visualized on nine discriminant functions. Each point represents the positioning of each MHC allele within the first nine discriminant functions. The circles represent MHC supertypes. The inset graph shows the number of discriminant functions that were used for this DAPC. (b) Total number of MHC (i) alleles (Ai) and (ii) supertypes (STi) in individual fish in the ornamental and wildtype populations. This box and whisker plot indicates the median (dark line), the highest and lowest observations (vertical lines from the box) and the ourliers (dots)

A total of 66 MHC alleles and 13 supertypes were identified in all the fish analysed in this study (Figure 2a). Guppies possess multiple MHC class IIB loci, and the number of alleles differed between the wildtype and ornamental fish. Wildtype fish had a significantly higher number of MHC alleles per individual (*Ai*) (mean ± SE *Ai* = 2.60 ± 0.07) compared to ornamental fish (*Ai* = 1.73 ± 0.04) (*t*_1736_ = 12.01, *p* ≤ .001; Figure 2b.i). The number of MHC supertypes per individual (*STi*) was also significantly higher in wildtype compared to ornamental fish (mean ± SE *STi* = 1.82 ± 0.05, and 1.52 ± 0.02 respectively) (*t*_1736_ = 6.74, *p* ≤ .001; Figure 2b.ii).

### 3.3 Host MHC supertype and parasite intensity

**FIGURE 3:**
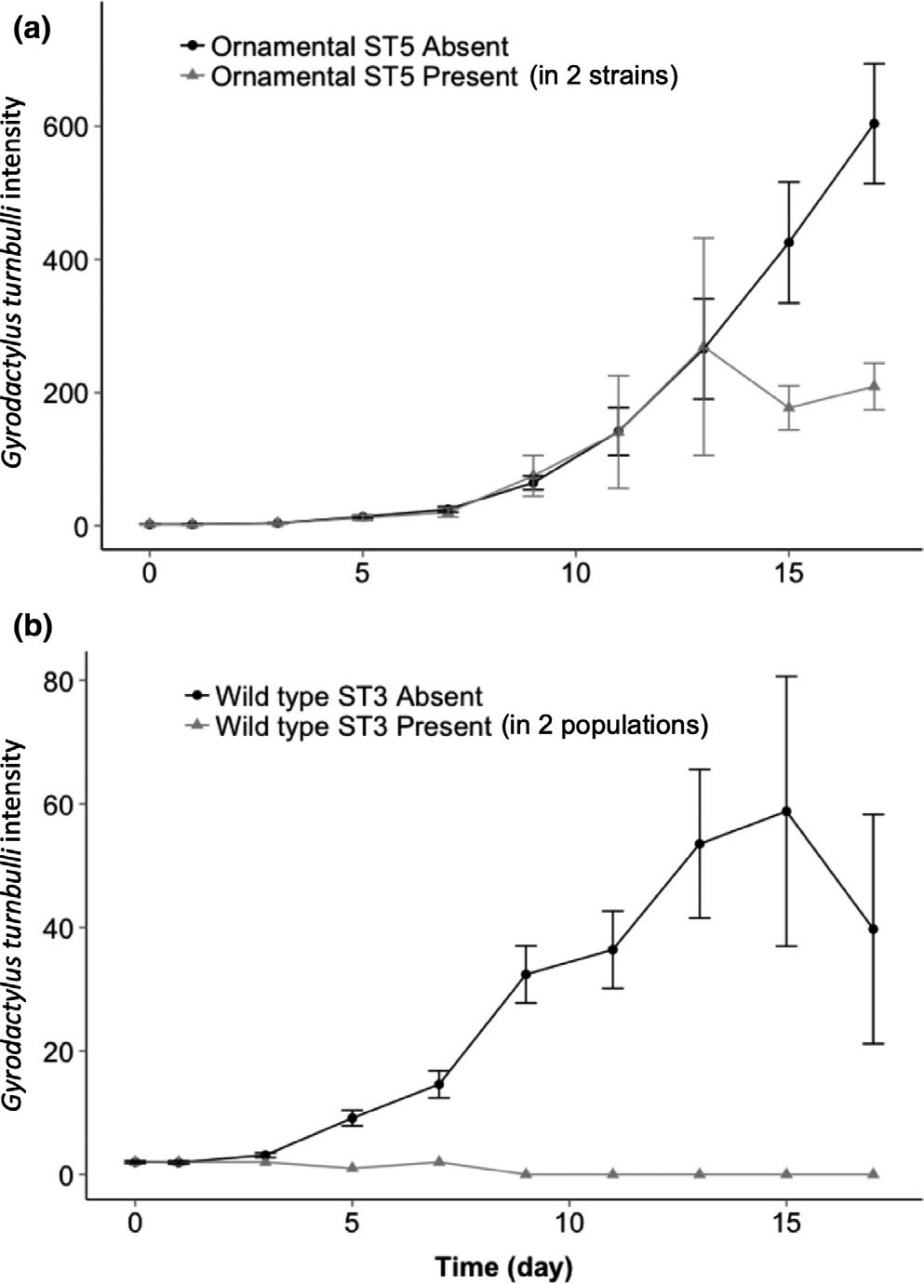
Total *Gyrodactylus turnbulli* intensity on (a) ornamental and (b) wildtype guppies (*Poecilia reticulata*) over time in the presence (grey) and absence (black) of a major histocompatibility complex allele from (a) supertype 5 and (b) supertype 3. Bars represent standard error of the mean. The association between the presence of a given supertype and *Gyrodactylus turnbulli* intensity is not driven by a single strain; supertype 5 is present in two ornamental strains (Red, *n* = 93) and Black, *n* = 12), and supertype 3 is present in both the Lower Aripo (*n* = 38) and the Tacarigua (*n* = 59)

The parasite intensity on ornamental guppies was significantly lower on fish with MHC genotypes containing alleles of supertype ST5 (GLMM: *z*_1421_ = −4.30, *p* ≤ .001, Figure 3a; Figure S3). Supertype ST12 had a marginally significant effect on parasite intensity on these ornamental fish (*z*_1421_ = −2.11, *p* = .035; Figure S4). Wildtype fish parasite intensity was significantly lower on fish with ST3 (GLMM: *z*_1133_ = −3.31, *p* ≤ .001, Figure 3b), and also here, one supertype (ST7) showed a marginal effect (*z*_1133_ = 2.10, *p* = .036; Figure S4b). Hereafter, we refer to ST5 and ST12 as the “resistant” supertypes in the ornamental lines, and ST3 and ST7 as the “resistant” supertypes in the wildtype. Allelic diversity, supertype diversity and the standard length of individual guppies did not significantly affect parasite intensity in wildtype or ornamental fish (*p* > .05).

**FIGURE 4:**
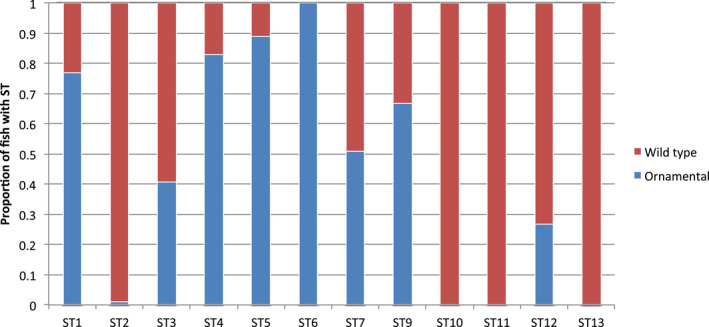
Proportion of wildtype (red) and ornamental (blue) guppies (*Poecilia reticulata*) with each major histocompatibility complex class II supertype (ST) at the population level

The presence of ST5 and ST12 in the ornamental guppies, and ST3 and ST7 in the wildtype guppies is associated with a significantly reduced *G. turnbulli* load. It is possible that the “resistant” supertypes are more common in the population where they are seen to offer resistance, which could result in a stronger statistical power to detect such an association. Figure 4 shows there are differences in relative frequencies of STs between the wildtype and ornamental lines, which makes this a distinct possibility. A (mutually nonexclusive) hypothesis is that the “resistant” supertypes of the ornamental and wildtype populations share molecular similarities which offer these populations better protection against *G. turnbulli* infection. Both ST5 and ST3 appear to have the potential to confer *G. turnbulli* resistance, and hence we hypothesized that they may share a number of amino acids (AAs) that are unique to these supertypes. Using our genetic algorithm, two amino acid (AA) positions in the PBR that were shared by all the alleles of ST5 and ST3 were detected, making them unique from the alleles of all other STs. When AA12 = *N* (asparagine) or AA15 = *M* (methionine), the ST is correctly assigned to either ST3 or ST5. Conversely, alleles without AA12 = N and AA15 = M do not belong to ST3 or ST5 in all but two cases. The analysis with our genetic algorithm revealed that the “resistant” supertypes share similarities in their physicochemical properties of the amino acids that comprise their PBR, which make them unique from all other STs.

## 4 DISCUSSION

The current study highlights that breeding stocks in aquaculture are prone to lose immunogenetic diversity, and that this loss could significantly compromise the host's resistance to parasites. We assessed the MHC) class II genotypes and immunocompetence of a model fish species, the guppy *Poecilia reticulata*, comparing recent descendants of wild caught guppies (“wildtype” guppies) with guppies from the pet trade that have been selectively bred over decades (“ornamental” guppies). The number of MHC alleles and MHC supertypes per individual was significantly lower in ornamental guppies than in wildtype guppies. In addition, also at the population level, the ornamental lines had lower supertype diversity than the wildtype populations, similar to previous studies of neutral genetic diversity (Bleakley et al., 2008; Shen et al., 2007). The ornamental guppies had lost three out of 13 supertypes, and in the wildtype fish only one supertype was absent. Furthermore, ornamental fish were significantly more susceptible to *Gyrodactylus turnbulli* infections, accumulating a 10‐fold higher intensity at the peak of the infection compared to their wildtype counterparts. Variation in parasite intensity was linked to the presence of certain MHC supertypes. This difference was so pronounced that the worm intensity differed by several orders of magnitude, depending on whether the host was a carrier of a “resistant” supertype or not. Surprisingly, this analysis showed that the supertypes that were associated with resistance to *G. turnbulli* infections were not the same for ornamental and wildtype guppies, and we identified two amino acid substitutions in the MHC PBR that are strongly linked to resistance.

Previous studies have shown that specific MHC genotypes are associated with parasitic intensity (Ditchkoff et al., 2005; Paterson et al., 1998) and that intermediate MHC allelic diversity leads to a greater pathogen resistance than those with a low or high diversity (Wegner et al., 2003). The current study found that the presence of alleles from particular functional clusters (supertypes) is significantly linked to an individual's parasite susceptibility, over the level of MHC allelic diversity; this is consistent with Schwensow et al. (2007), Fraser and Neff (2010) and Wang et al. (2017). Fraser and Neff (2010) identified “types” of MHC alleles based on unique PBRs, showing that the presence of a particular “type” significantly reduced *Gyrodactylus* intensity. Only few of the MHC alleles identified by Fraser and Neff (2010) were present in the current study (*n* = 12) and those that were shared were not from the same “type” group, hindering direct comparison. The method we used to determine the supertype groups was also different to that used by Fraser and Neff (2010). They grouped according to allele “type” associating alleles that differed by three amino acids or fewer that had unique peptide binding residues, whereas we focused on the five most common allele types; all other types were grouped into a “rare” category. Unfortunately, this hinders a direct comparison between the two studies.

In the ornamental strains, ST5 and ST12 were associated with increased resistance, whereas ST3 and ST7 were associated with resistance in the wildtype fish. Interestingly, these supertypes share similarities in their physicochemical properties of the amino acids that comprise their PBR. Using our genetic algorithm, we show that alleles assigned to ST5 and ST3 share two amino acid (AA) positions in the PBR that are unique to both supertypes. Irrespective of whether a supertype belongs to an ornamental or wildtype strain, guppies are significantly more resistant if they possess one MHC allele with either an asparagine at AA position 12 in the PBR or a methionine at AA position 15. The ornamental and wildtype populations also differed in relative frequency of these supertypes. It is therefore possible that the more common supertype (i.e., ST5 in the ornamental, and ST3 in the wildtype) possessed greater statistical power for detecting an association with resistance. This might explain why the analyses picked out different “resistant” supertypes in these two populations. Altogether, these data show that the supertype demarcation captures some but not all of the variation in the immune function of the alleles. This implies that the immunological function of a given supertype can be partially covered by another supertype, albeit with less efficiency. Consequently, the near loss of one of the “resistant” supertypes (ST3) in the ornamental strains (which went down from 10.2% in the wildtype to 2.9% in the ornamental fish) is partially compensated for by the increase in frequency of the second “resistant” supertype (ST5) (which went up from 12.4% in the wildtype to 39.8% in the ornamental guppies). The latter supertype (ST5) might be less efficient than ST3 in fighting off the gyrodactylid infection, which could explain why the ornamental fish have a considerably higher parasite load on average compared to the wildtype guppies.

The second main finding is that the ornamental guppies have an inferior immune response to *G. turnbulli* infection compared to the wildtype fish. It is likely that wildtype and ornamental fish differ more widely across the genome, and these effects are not analysed in our study. Fish stocks in aquaculture are at risk of losing functionally important immunogenetic variation due to a number of significant changes in the evolutionary forces that act on these genomes in captivity. Compared to natural populations, captive genepools of livestock are often under strong directional selection for a small number of desirable traits such as growth rate or yield. Directional selection can erode variation across the genome by reducing the effective population size (*Ne*) and increasing genetic drift (Charlesworth, 2009). For example, the GIFT (Genetically Improved Farmed Tilapia) strain of Nile tilapia (*Oreochromis niloticus*) was founded early this millennium, and its selection programme aimed to improve growth rate whilst avoiding inbreeding. Nevertheless, the GIFT strain has an estimated *Ne* < 100 individuals (Ponzoni et al., 2010), which will result in a significant amount of drift and loss of immunogenetic variation. Artificial selection on a relatively small founder genepool is likely to be common in aquaculture, and significant effects of inbreeding have been recorded in various aquatic animal species, including Atlantic salmon, brook trout, chinook salmon, coho salmon, rainbow trout and Nile tilapia (Fessehaye et al., 2009; Fjalestad, 2005). Our data show that during the domestication/selective breeding of ornamental guppies, functionally important supertype variation of the MHC has been lost, which is significantly associated with a compromised immunocompetence of these fish. It is not unlikely that various brood stocks of economically important fish species used in aquaculture may have experienced a similar decline in immunocompetence. Our research emphasizes the importance of maintaining genetic diversity in aquaculture stocks, as has been identified by the Convention on Biological Diversity (CBD), and in particular by its Strategic goal C, Aichi Target 13 (CBD, 2010).

Not all species may suffer the same amount from founder effects, inbreeding or artificial selection during domestication. For example, several big cat populations that have undergone extreme bottlenecks retain functional supertype diversity despite low MHC allelic diversity (Schwensow et al., 2019). In some fish species, such as the self‐fertilizing fish *Kryptolebias marmoratus*, robustness against inbreeding has evolved by changing the genetic architecture of the MHC and spreading polymorphisms across many duplicated loci (Ellison et al., 2012; van Oosterhout, 2013; Sato et al., 2002). However, the deleterious impact of these processes is particularly pronounced for loci with high levels of gene diversity, such as the MHC of guppies. In guppies, 13 supertypes segregate at an estimated two to three MHC class II loci (van Oosterhout et al., 2006), which implies that multiple supertypes segregate at the same genetic locus (Lighten et al., 2017). Consequently, MHC diversity is lost because genetic drift results in the fixation (and hence loss) of some of these supertypes (as well as that of other immune genes), which can explain compromised immunocompetence of the ornamental guppy strains. With further development of genome editing (Kim & Kim, 2014), it may become possible in the future to construct haplotypes of commercially important fishes consisting of a series of orthologous gene copies that represent different, functionally important supertypes. This would be similar to the genetic architecture of *K. marmoratus* MHC, which is resilient against even the most severe inbreeding. Here we highlight that artificial selection of economically important traits can lead to loss of key immune functional alleles that protect populations from the risk of parasitic infection.

## CONFLICTS OF INTEREST

The authors have no conflicts of interest.

## AUTHOR CONTRIBUTIONS

J.C., C.vO. and W.S. designed the study; W.S. performed all experiments; W.S. and A.E. analysed the data with S.P. providing the genetic algorithm of supertypes; W.S. drafted the paper with contributions from J.C., A.E. and C.vO.

## Supporting information

Supplementary Material

## Data Availability

Raw amplicon sequence data associated with this article are deposited on NCBI SRA under accession PRJNA520831.
