# Supplementary material for: Depletion of MHC supertype during domestication can compromise immunocompetence
Source: Mol Ecol. 2020 Dec 22;30(3):736–46. doi: 10.1111/mec.15763 (PMC7898906; doi:10.1111/mec.15763)
Supplement: Supplementary file 1 — Supplementary Material [file MEC-30-736-s001.docx]

**Supplemental Information for:**

**Depletion of MHC supertype during domestication can**

**compromise immunocompetence**

Smallbone W, Ellison A, Poulton S, van Oosterhout C and Cable J.

**Table of Contents:**

| **S1: Supplementary materials and methods** | **Page 2-4** |
| --- | --- |
| **S2: Supporting justification for data analysis** | **Page 4** |
| **S3: Genetic Algorithm** | **Page 5-6** |
| **Table S1: Effect of the interaction between each guppy**  **(*Poecilia reticulata*) strain and time (day) on parasite**  **(*Gyrodactylus turnbulli*) intensity** | **Page 7-8** |
| **Figure S1: Rate of change in Bayesian information criterion**  **with additional clusters when using discriminant analysis**  **of principle components of Major Histocompatibility**  **Complex class II alleles from *Poecilia reticulata*** | **Page 9** |
| **Figure S2: Nearest neighbour phylogenetic tree of the**  **relationship of guppy (*Poecilia reticulata*) Major**  **Histocompatibility Complex alleles** | **Page 10** |
| **Figure S3: Parasite (*Gyrodactylus turnbulli*) intensity over**  **time (days) on ten strains of guppy (*Poecilia reticulata*)** | **Page 11** |
| **Figure S4: Total *Gyrodactylus turnbulli* intensity on**  **(a) ornamental and (v) wildtype guppies (*Poecilia reticulata*)** | **Page 12** |

**S1: Supplementary** **materials and methods**

Initially, the number of clusters (k) were identified using 5000 repeats of *find.clusters* function for *k* = 1-30, with arguments n.iter = 5000 and n.start = 500, and retaining all, 100, principal components (PCs; following methods as Phillips et al. 2018). MHC alleles are clustered into 13 supertype groups based on the amino acid sequence of the peptide-binding region where there is a drop off in rate of increase of change in the Bayesian Information Criterion (ΔBIC; (**Fig. S1**). Adding additional clusters increased the risk that iteration would not have 13 clusters and the rate of increase of ΔBIC on adding additional clusters dramatically reduced (**Fig. S1**). The discriminant analysis of principle components (DAPC) identifies the probability of an allele being in a particular supertype based on the retained discriminant functions, using 30 principle components above which little information is gained with 13 discriminant function eigenvalues due to the low cluster number. The optimal number of discriminant functions to retain was identified by performing k means clustering with k =13 PCs = 30, using the package *adegenet* and the function *optim.a.score* nine discriminant functions were determined optimal. Clusters for which the amino acid sequence of each MHC class II allele was assigned, were analysed through DAPC at 1000 iterations with the cluster names standardized between runs (**Fig. 2a**). Allele sequences were assigned their model supertype cluster. An additional run of 1000 iterations of DAPC was performed and compared to the initial run of DAPC for repeatability using a mantel test in the package *vegan* (99% consistency between runs). The consistency of each individual alleles cluster membership indicates distinctiveness of the clusters. DAPC clustering was repeated 2000 times and alleles supertype classification was used when consistently in 60% or over of the 2000 runs of DAPC analysis. Alleles that were not consistent in their supertype clustering were removed from downstream analysis (n = 33, 13%).

All statistical analyses were conducted using R version 2.15.1 (version 1.0.136, RStudio 2009-2016 RStudio, Inc.).

As parasite intensity, feeding lunges and time were recorded for each individual fish at different time points, ‘Fish ID’ was included as a random effect in all of the Generalised Linear Mixed Models (GLMMs) to avoid pseudo-replication, by incorporating repeated-measures. The residuals of all of the models were normally distributed. Only significant terms are reported.

Variation in parasite intensity, defined as the number of worms on an infected host (Bush et al. 1997), between wildtype and ornamental strains of fish was analysed used a GLMM (*glmmadmb* package), with negative binomial distribution and “log” link function. The starting GLMM used parasite intensity as the response variable, with the explanatory variables: number of days post infection (day); host standard length; whether the host was a wild or ornamental strain; interaction host standard length × wildtype/ornamental and the interaction day × wildtype/ornamental. Model refinement using the drop1 Akaike’s Information Criterion (AIC) methods suggested that the starting model was the most robust.

Variation in feeding rate between wild and ornamental fish and parasite intensity was assessed using a GLMM (*lme4* package), with the function glmer, gaussian family and “log” link function. The starting model included the explanatory variables: whether the host was wild or ornamental; host standard length; parasite intensity; and number of days from infection. The interactions day × wildtype/ornamental, parasite intensity × day, wildtype/ornamental × host standard length and parasite intensity × wildtype/ornamental were also included in the initial model. Using the drop1 function, the starting model was deemed the most robust model and all explanatory variables remained for the analysis. A GLMM was run to assess the variation in the feeding rate between wildtype and ornamental fish and infected and uninfected individuals, using the Gamma family with the “inverse” link function. The initial model included as explanatory variables: whether the host was wildtype or ornamental, host standard length, infection status (treatment), and number of days from infection. The interactions day × wildtype/ornamental, treatment × day, treatment × wildtype/ornamental, host standard length × wildtype/ornamental and the three way interaction of treatment × wildtype/ornamental × day were also included in the initial model. Using the drop1 model refinement function the starting model was identified as the most robust, leaving all explanatory variables in the final model.

Differences in MHC genotype and supertype community across type of host (wildtype and ornamental) were visualised through non-metric multidimensional scaling (NMDS), using the metaMDS function (*vegan* package) (Oksanen et al. 2007). Non-metric multidimensional scaling is an ordination method used to measure dissimilarity measures (function vegdist) by running NMDS several times from a random starting configuration, comparing outputs (function procrustes) then stops after finding a similar minimum stress solution twice. The ordination was run for 1000 iterations; with stress scores of 0.04 for allele community and 0.06 for supertype community, the final solution was sufficiently low to enable reliable interpretation in two dimensions. Effect of host type on allele and supertype were assessed using the manyglm function (*mvabund* package; Wang et al. 2012). The method computes the analysis of deviance for a multivariate GLM, fitting a single GLM to each response variable with a common set of predictors. Monte-Carlo resampling tested for a significant community level response to the predictors. Analysis was conducted with (i) allele and (ii) supertype community as the dependent variable being explained by host type (wildtype or ornamental) and strain of host. Model refinement was performed using the function drop1, which identified that both explanatory variables should remain in the model. To identify differences in the number of alleles and supertype per individual (*Ai* and *STi*, respectively) between wildtype and ornamental fish, an Anova test (lm function in base R) was performed.

To assess the effect of supertypes on parasite intensity, a GLMM was performed using the package *glmmadmb* with the family negative binomial. The starting model included the explanatory variables experimental day, host standard length, host allelic diversity and host supertype diversity and the dependent variable *G. turnbulli* intensity. The data were subset into wildtype and ornamental fish due the presence of some supertypes in one of these host types; a significant difference between these two groups of fish was identified in the previous analysis, justifying the sub-setting (**Supplementary S1**). It was not possible to include all of the biologically relevant explanatory variables in the starting model without sub-setting. Microsoft Excel was used to determine the binomial distribution probability (binomdist) of MHC supertypes between wildtype and ornamental fish; the results suggested a significant difference giving additional reason for data sub-setting (**Fig. S2**). The additional explanatory variables included for (1) ornamental fish: ST3, ST4, ST5, ST7 and ST12; and (2) wild fish: ST3, ST4, ST5, ST7, ST12 and ST13, the interaction between each of these supertypes and experimental day was also included in the model to identify if the supertype functional cluster elicited an immune response. A random term of “Fish ID” was also included to account for pseudo-replication. The models were refined using drop1 AIC refinement. The starting models for both analyses were the most robust and so all explanatory variables were retained for the analysis.

**S2: Supporting justification for data analysis.** The effect of splitting data into two subsets (wildtype and ornamental) compared to the entire date set. The model explained the variation in *Gyrodactylus turnbulli* intensity on guppies (*Poecilia reticulata*) using the explanatory variables time (day); host standard length; number of MHC alleles per individual (*Ai*); number of MHC supertypes per individual (*STi*); the presence of: supertype 3, supertype 4, supertype 5, supertype 7, supertype 12; and the interaction between each supertype × time (day). These variables were selected as those that were present in both subsets of data. The results of this analysis showed that the model with all fish was not significantly different from the results for wildtype fish but was significantly different to the ornamental subset of data (p = 1; p ≤ 0.001. respectively).

**S3: The logical model described here is based on a generic model under development by BioEcoSS Ltd, as applied specifically to the data analysed in this paper**.

1. The analysis dataset comprises a two-dimensional matrix of aligned amino acid sequences in rows. The first column is a numeric code for the supertype, followed by n columns of alpha codes for amino acids. Thereafter:
2. Define the supertype as the target variable and define one supertype code as the target value.
3. Define all other columns as predictor variables.
4. Optionally define filters, subsets and parameters for the analysis. Filters and subsets allow discrete analyses to be carried out on subsets of the data, primarily to allow different target values to be assessed. User-defined parameters include population size and generations, plus randomisation probabilities for operators, conjunctions, *etc*.
5. The algorithm starts by generating a “founder population” of logical expressions (predictive rules), based on a randomly chosen predictor variable and a randomly chosen value, of the form; “AA1 = A”. The size of the population is user-defined but is usually in the order of 10 – 20.
6. Each of these predictive rules is tested against the target expression by tallying a 2 x 2 contingency table. This creates four cells: Target true/Prediction true, Target false/Prediction false, both of which constitute correct predictions, and two cells representing incorrect predictions. The phi (φ) coefficient is used to score this contingency table:

$$=\frac{f_{11}f_{00}-f_{10}f_{01}}{\sqrt{C_{1}C_{2}R_{1}R_{2}}}$$

where *f* = frequency in the relevant cell, *C* = the relevant column total and *R* = the relevant row total.

1. The n top scoring predictive rules (n is user-defined – usually 10) are saved as the “breeding population” and any remaining rules are killed off.
2. Each rule in the breeding population is then used to breed a new rule through a number of different operations, selected randomly according to user-defined probabilities;
   - Altering the value.
   - Changing the operator.
   - Combining rules using AND or OR conjunctions.
   - Modifying existing combinations by changing the conjunction or removing elements.
   - Stochastically creating entirely new predictive rules.
3. All rules (parents and children) are scored using the process defined in Stage 6. The phi coefficient is penalised (a user-defined value, default 2%) by the number of variables employed in each rule to ensure a parsimonious rule structure.
4. Cycle through Stages 7 & 8 for n generations, (n is user-defined – usually 5,000 or 10,000). A user-defined stopping rule can be applied to halt execution after a given number of generations with no improvement in the top score.
5. The whole process from Stages 5 to 10 is run n times (n is user-defined– usually 5 or 10) to generate multiple populations. These are necessary to overcome local bottlenecks or “extinctions”, and to explore different regions of multi-dimensional space.

**Table S1:** Effect of the interaction between each guppy (*Poecilia reticulata*) strain and time (day) on parasite (*Gyrodactylus turnbulli*) intensity. Wildtype strains of fish are reported. Significant results are highlighted grey.

| **Strain × Day** | **Compared to** | ***D.f.*** | ***Z*** | ***P*** |  |
| --- | --- | --- | --- | --- | --- |
| Balcony × Day | Black | 9 | -0.11 | 0.916 |  |
|  | Blonde Red | 9 | -1.47 | 0.143 |  |
|  | Cobra Green | 9 | 0.41 | 0.685 |  |
|  | Flame | 9 | 1.25 | 0.211 |  |
|  | Lower Aripo (wildtype) | 9 | -4.12 | ≤0.001 |  |
|  | Neon Blue | 9 | -0.07 | 0.947 |  |
|  | Sunset Blonde | 9 | 1.12 | 0.262 |  |
|  | Tacarigua (wildtype) | 9 | -5.06 | ≤0.001 |  |
|  | Yellow German | 9 | 0.48 | 0.632 |  |
| Black × Day | Blonde Red | 9 | -1.84 | 0.065 |  |
|  | Cobra Green | 9 | 0.68 | 0.495 |  |
|  | Flame | 9 | 1.68 | 0.092 |  |
|  | Lower Aripo (wildtype) | 9 | -5.03 | ≤0.001 |  |
|  | Neon Blue | 9 | 0.05 | 0.959 |  |
|  | Sunset Blonde | 9 | 1.69 | 0.091 |  |
|  | Tacarigua (wildtype) | 9 | -6.80 | ≤0.001 |  |
|  | Yellow German | 9 | 0.79 | 0.430 |  |
| Blonde Red × Day | Cobra Green | 9 | 2.95 | 0.003 |  |
|  | Flame | 9 | 3.65 | ≤0.001 |  |
|  | Lower Aripo (wildtype) | 9 | -4.11 | ≤0.001 |  |
|  | Neon Blue | 9 | 2.00 | 0.045 |  |
|  | Sunset Blonde | 9 | 4.55 | ≤0.001 |  |
|  | Tacarigua (wildtype) | 9 | -6.41 | ≤0.001 |  |
|  | Yellow German | 9 | 3.18 | 0.002 |  |
| Cobra Green × Day | Flame | 9 | 1.19 | 0.235 |  |
|  | Lower Aripo (wildtype) | 9 | -6.11 | ≤0.001 |  |
|  | Neon Blue | 9 | -0.66 | 0.512 |  |
|  | Sunset Blonde | 9 | 1.09 | 0.276 |  |
|  | Tacarigua (wildtype) | 9 | -8.68 | ≤0.001 |  |
|  | Yellow German | 9 | 0.10 | 0.921 |  |
| Flame × Day | Lower Aripo (wildtype) | 9 | -6.38 | ≤0.001 |  |
|  | Neon Blue | 9 | -1.69 | 0.091 |  |
|  | Sunset Blonde | 9 | -0.39 | 0.697 |  |
|  | Tacarigua (wildtype) | 9 | -8.24 | ≤0.001 |  |
|  | Yellow German | 9 | -1.13 | 0.258 |  |
| Lower Aripo (wildtype) × Day | Neon Blue | 9 | 5.24 | ≤0.001 |  |
|  | Sunset Blonde | 9 | 7.43 | ≤0.001 |  |
|  | Tacarigua | 9 | -0.43 | 0.668 |  |
|  | Yellow German | 9 | 6.33 | ≤0.001 |  |
| Neon Blue × Day | Sunset Blonde | 9 | 1.72 | 0.086 |  |
|  | Tacarigua (wildtype) | 9 | -7.24 | ≤0.001 |  |
|  | Yellow German | 9 | 0.77 | 0.443 |  |
| Sunset Blonde × Day | Tacarigua (wildtype) | 9 | -11.13 | ≤0.001 |  |
|  | Yellow German | 9 | -1.02 | 0.307 |  |
| Tacarigua (wildtype) × Day | Yellow German | 9 | 9.16 | ≤0.001 |  |


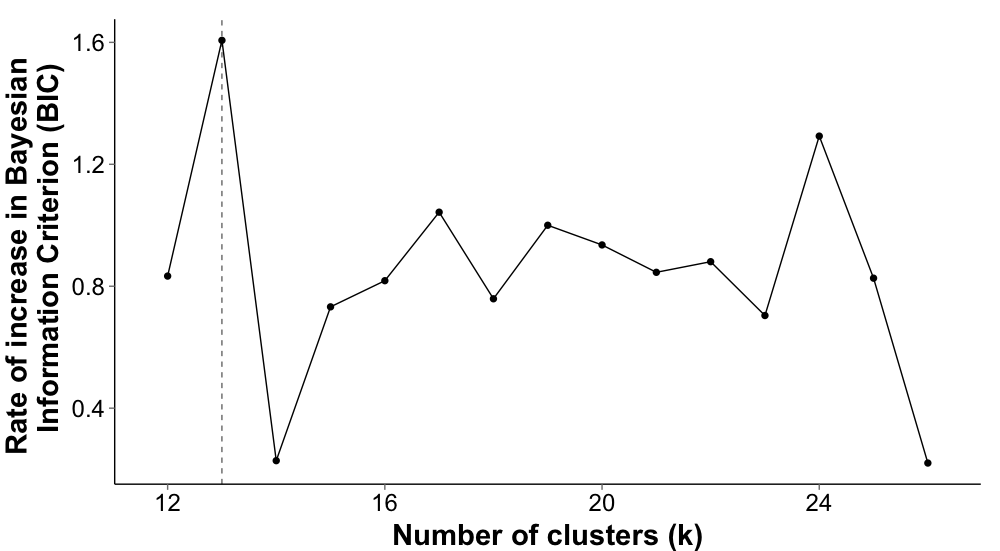


**Figure S1:** Rate of change in Δ Bayesian information criterion (BIC) with additional clusters (k) when using discriminant analysis of principle components of Major Histocompatibility Complex class II alleles from *Poecilia reticulata*. Dotted line shows the peak at 13 clusters.

**Figure S2:** Nearest neighbour phylogenetic tree of the relationship of guppy (*Poecilia reticulata*) Major Histocompatibility Complex alleles (identified from the current study and published). Alleles are coloured based on their inferred clustering to 13 supertypes (ST) inferred through Discriminant Analysis of Principle Components (DAPC).

**ST1**

**ST2**

**ST3**

**ST4**

**ST5**

**ST6**

**ST7**

**ST8**

**ST9**

**ST10**

**ST11**

**ST12**

**ST13**

**NA**

**
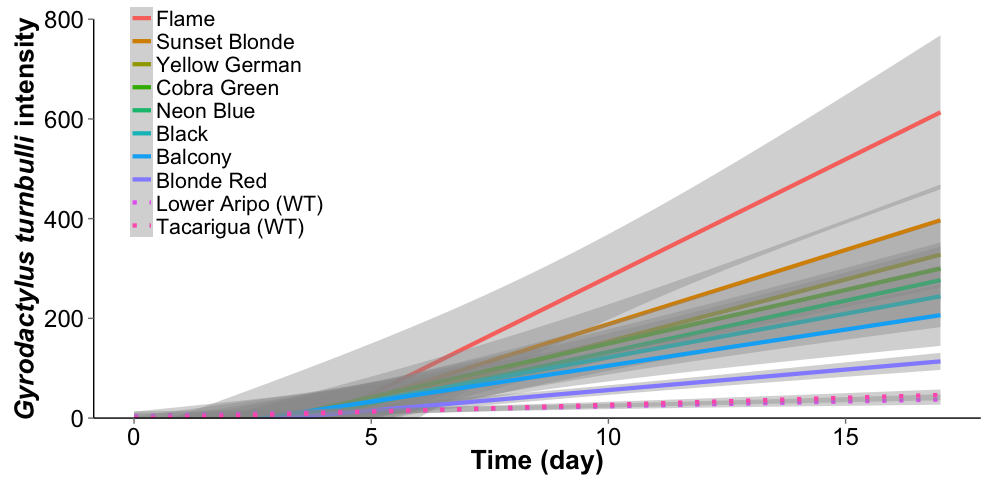
**

**Figure S3:** Parasite (*Gyrodactylus turnbulli*) intensity over time (days) on ten strains of guppy (*Poecilia reticulata*). Wildtype (WT) strains are dotted, whilst ornamental strains are solid. Grey shading represents standard error.


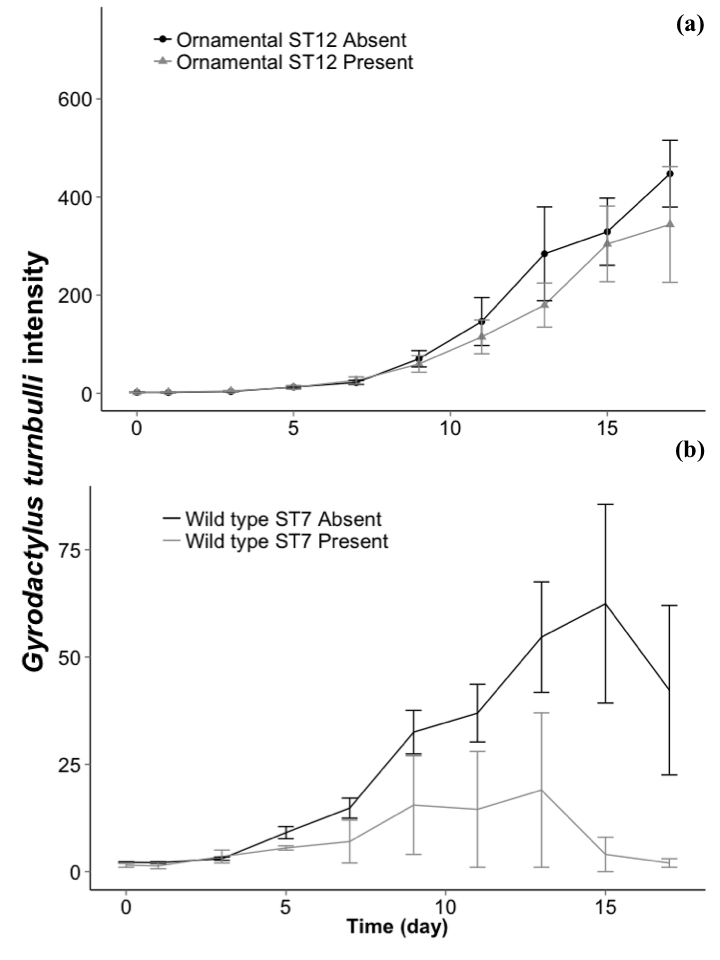


**Figure S4:** Total *Gyrodactylus turnbulli* intensity on (a) ornamental and (v) wildtype guppies (*Poecilia reticulata*) over time in the presence (grey) and absence (black) of a Major Histocompatibility Complex allele from (a) supertype 12 and (b) supertype 7. Bars represent standard error of the mean.
